# Association between the timing of ICU admission and mortality in patients with hospital-onset sepsis: a nationwide prospective cohort study

**DOI:** 10.1186/s40560-023-00663-6

**Published:** 2023-04-21

**Authors:** Yoon Hae Ahn, Jinwoo Lee, Dong Kyu Oh, Su Yeon Lee, Mi Hyeon Park, Haein Lee, Chae-Man Lim, Sang-Min Lee, Hong Yeul Lee, the Korean Sepsis Alliance (KSA) Investigators, Chae-Man Lim, Sang-Bum Hong, Dong Kyu Oh, Gee Young Suh, Kyeongman Jeon, Ryoung-Eun Ko, Young-Jae Cho, Yeon Joo Lee, Sung Yoon Lim, Sunghoon Park, Jeongwon Heo, Jae-myeong Lee, Kyung Chan Kim, Yeon Joo Lee, Youjin Chang, Kyeongman Jeon, Sang-Min Lee, Chae-Man Lim, Suk-Kyung Hong, Woo Hyun Cho, Sang Hyun Kwak, Heung Bum Lee, Jong-Joon Ahn, Gil Myeong Seong, Song-I. Lee, Tai Sun Park, Su Hwan Lee, Eun Young Choi, Jae Young Moon

**Affiliations:** 1 Department of Critical Care Medicine, Seoul National University Hospital, 101 Daehak-Ro, Jongno-Gu, Seoul, 03080 Korea; 2 Division of Pulmonary and Critical Care Medicine, Department of Internal Medicine, Seoul National University Hospital, Seoul, Korea; 3 Department of Pulmonary and Critical Care Medicine, Asan Medical Center, Seoul, Korea

**Keywords:** Sepsis, Prognosis, Patient admission, Intensive care unit, Septic shock, Transfer

## Abstract

**Background:**

Based on sparse evidence, the current Surviving Sepsis Campaign guideline suggests that critically ill patients with sepsis be admitted to the intensive care unit (ICU) within 6 h. However, limited ICU bed availability often makes immediate transfer difficult, and it is unclear whether all patients will benefit from early admission to the ICU. Therefore, the purpose of this study was to determine the association between the timing of ICU admission and mortality in patients with hospital-onset sepsis.

**Methods:**

This nationwide prospective cohort study analyzed patients with hospital-onset sepsis admitted to the ICUs of 19 tertiary hospitals between September 2019 and December 2020. ICU admission was classified as either early (within 6 h) or delayed (beyond 6 h). The primary outcome of in-hospital mortality was compared using logistic regression adjusted for key prognostic factors in the unmatched and 1:1 propensity-score-matched cohorts. Subgroup and interaction analyses assessed whether in-hospital mortality varied according to baseline characteristics.

**Results:**

A total of 470 and 286 patients were included in the early and delayed admission groups, respectively. Early admission to the ICU did not significantly result in lower in-hospital mortality in both the unmatched (adjusted odds ratio [aOR], 1.35; 95% confidence interval [CI], 0.99–1.85) and matched cohorts (aOR, 1.38; 95% CI, 0.94–2.02). Subgroup analyses showed that patients with increasing lactate levels (aOR, 2.10; 95% CI, 1.37–3.23; *P* for interaction = 0.003), septic shock (aOR, 2.06; 95% CI, 1.31–3.22; *P* for interaction = 0.019), and those who needed mechanical ventilation (aOR, 1.92; 95% CI, 1.24–2.96; *P* for interaction = 0.027) or vasopressor support (aOR, 1.69; 95% CI, 1.17–2.44; *P* for interaction = 0.042) on the day of ICU admission had a higher risk of mortality with delayed admission.

**Conclusions:**

Among patients with hospital-onset sepsis, in-hospital mortality did not differ significantly between those with early and delayed ICU admission. However, as early intensive care may benefit those with increasing lactate levels, septic shock, and those who require vasopressors or ventilatory support, admission to the ICU within 6 h should be considered for these subsets of patients.

**Supplementary Information:**

The online version contains supplementary material available at 10.1186/s40560-023-00663-6.

## Introduction

Sepsis is characterized by life-threatening organ dysfunction caused by a dysregulated host response to an infection and is associated with high mortality [1]. Despite the advances made in clinical practice, the overall sepsis-associated hospital mortality rate remains high at 50.2 deaths per 100,000 in the United States [2]. Patients with sepsis often require treatment in the intensive care unit (ICU), and previous studies have reported that one-third of all sepsis deaths occur within 3 days of ICU admission [3]. As such, early identification of clinical deterioration and appropriate management are needed to improve outcomes [4].

In the ICU, tools for organ support in the form of mechanical ventilation, continuous renal replacement therapy (RRT), and extracorporeal life support are available, along with monitoring devices and higher staff-to-patient ratios to allow for the specialized management of critically ill patients. The positive impact of ICU admission on patient survival was found to be most evident during the first 72 h of critical illness, suggesting that prompt admission to the ICU is needed for better outcomes [5, 6]. A previous study conducted by Mohr et al. [7] showed that delayed admissions of patients with sepsis from the emergency department (ED) were associated with decreased sepsis bundle compliance and increased mortality, ventilator duration, and ICU and hospital length of stay (LOS). In another study of 12,380 patients, the 90-day mortality rate of a subgroup of critically ill patients was reduced by 16.2% with early admission to the ICU [8]. Based on such findings, the Surviving Sepsis Campaign (SCC) guideline suggests that adults with sepsis who require ICU admission be admitted to the ICU within 6 h [4]. This guideline, however, does not consider the individual idiosyncrasies of each patient, and whether admission to the ICU within 6 h will benefit all patients with sepsis remains controversial [9].

Despite the advantages of early ICU admission, the demand for ICU beds often exceeds supply. The limited availability of resources can hinder immediate transfer to the ICU [10]. In addition, some studies show no clear relationship between delays in ICU admission and patient outcomes, indicating that early ICU admission may not be beneficial for all patients [11]. The evidence behind the SSC guideline to admit patients to the ICU within 6 h has primarily focused on a broad subset of critically ill patients, and studies specific to sepsis patients are lacking. Patients with sepsis have complex disease processes and present with a spectrum of illness severity that requires an individualized treatment approach, such as the individualization of hemodynamic monitoring, shock management, and the timing of ICU admission [9, 12]. Thus, this study investigated the association between the timing of ICU admission and mortality in patients with hospital-onset sepsis.

## Methods

### Study design and patient population

This nationwide, multicenter, prospective cohort study analyzed patients with sepsis belonging to the Korean Sepsis Alliance registry between September 1, 2019, and December 31, 2020. Nineteen tertiary or university-affiliated hospitals in South Korea participated in the Korean Sepsis Alliance, including 13 centers operating the rapid response system (RRS). Adult patients aged ≥ 19 years diagnosed with hospital-onset sepsis according to the Sepsis-3 definitions [13] admitted to the ICU during the study period were included, and follow-up was conducted until hospital discharge or death. Patients with sepsis admitted to the ICU directly from the ED were ineligible for participation and were thus excluded. All data were anonymized to ensure individual privacy, and the institutional review boards (IRB) of all participating hospitals approved this study (approval number: IRB-H-1808-135-967). As an observational study, the decision to obtain or waive written informed consent was left to the discretion of the IRBs of the participating hospitals.

### Definitions

Hospital-onset sepsis was defined as sepsis diagnosed in the general ward, and time zero was the first time at which a patient was diagnosed with sepsis by the RRS. The activation criteria of the RRS at each participating hospital are shown in Additional file 1: Table S1. Septic shock was defined as a vasopressor requirement to maintain a mean arterial pressure (MAP) ≥ 65 mmHg with an initial serum lactate level > 2 mmol/L. Delta lactate was the difference in the lactate level from time zero to the time of ICU admission, and vasopressor use was the use of any one of the following: norepinephrine, vasopressin, epinephrine, and dopamine. Patients with a clinical frailty score ≥ 5 on the Clinical Frailty Scale [14] were classified as “frail.” Patients were classified into two groups based on the timing of ICU admission: those admitted to the ICU within 6 h were included in the early admission group, and those admitted beyond 6 h were included in the delayed admission group. The 6-h cutoff was used based on studies that showed that admission beyond this time was associated with worse outcomes and also based on the current sepsis guideline recommendation to admit patients to the ICU within 6 h [4, 15, 16].

### Outcome measures and subgroup analyses

The primary outcome was in-hospital mortality, and secondary outcomes were hospital LOS, ICU LOS, and discharge location. Six prespecified subgroup and interaction analyses for the primary outcome were performed according to the use of mechanical ventilation, vasopressors, or RRT on the day of ICU admission, delta lactate levels, malignancy, and clinical frailty status. A post-hoc subgroup analysis assessed whether the primary outcome differed according to the presence of septic shock on the day of ICU admission.

### Propensity score matching

Propensity scores were used to estimate the probability, based on baseline covariates, that patients would be admitted to the ICU within or beyond 6 h. Individual propensities were estimated with a logistic regression model using the following variables: age, sex, body mass index (BMI), comorbidities (cardiovascular disease, diabetes mellitus, chronic liver disease, chronic kidney disease, solid malignancy, hematologic malignancy, and chronic obstructive pulmonary disease), clinical frailty scores, sequential organ failure assessment (SOFA) scores, the presence of septic shock, and initial lactate levels. The standardized mean difference (SMD) was used to assess the balance between the two groups, and a difference < 0.1 was considered ideal [17]. After propensity score estimation, nearest neighbor matching without replacement using a caliper width equal to 0.2 of the standard deviation (SD) of the logit of the propensity score was performed to match the patients in the early and delayed admission groups in a 1:1 ratio [17–19].

### Statistical analysis

Categorical variables were expressed as counts and percentages, and continuous variables were reported as means and SD or medians and interquartile ranges (IQR). Between-group differences in baseline characteristics were assessed using the Student’s *t* test or the Mann–Whitney *U* test for quantitative variables and the Chi-square test or the Fisher exact test for qualitative variables. In the unmatched cohort, the primary outcome was assessed using logistic regression analysis adjusted for key prognostic factors (age and initial SOFA scores). The propensity score model and the outcome regression model were combined to construct a doubly robust estimator, which provides an estimation of the treatment effect for the primary outcome protected against possible model misspecification [20–22]. A sensitivity analysis using a logistic regression model adjusted for the center variable was performed to account for intercenter differences. Results were presented as odds ratios (OR) with corresponding 95% confidence intervals (CI). Kaplan–Meier survival curves were used to show cumulative mortality, and differences in survival were assessed using the log-rank test. Predefined subgroup analyses were performed for the primary outcome, and a formal test of interaction in a logistic regression model was used to assess whether the treatment effects showed significant differences between the subgroups. All analyses were two-tailed, and *P* values < 0.05 were considered to indicate statistical significance. Statistical analyses were performed using R Statistical Software (version 4.1.3; R Foundation for Statistical Computing, Vienna, Austria) and International Business Machine (IBM) Statistical Package for the Social Sciences (SPSS) Statistics (version 24.0 for Windows; IBM, Armonk, NY).

## Results

### Study participants

**Fig. 1:**
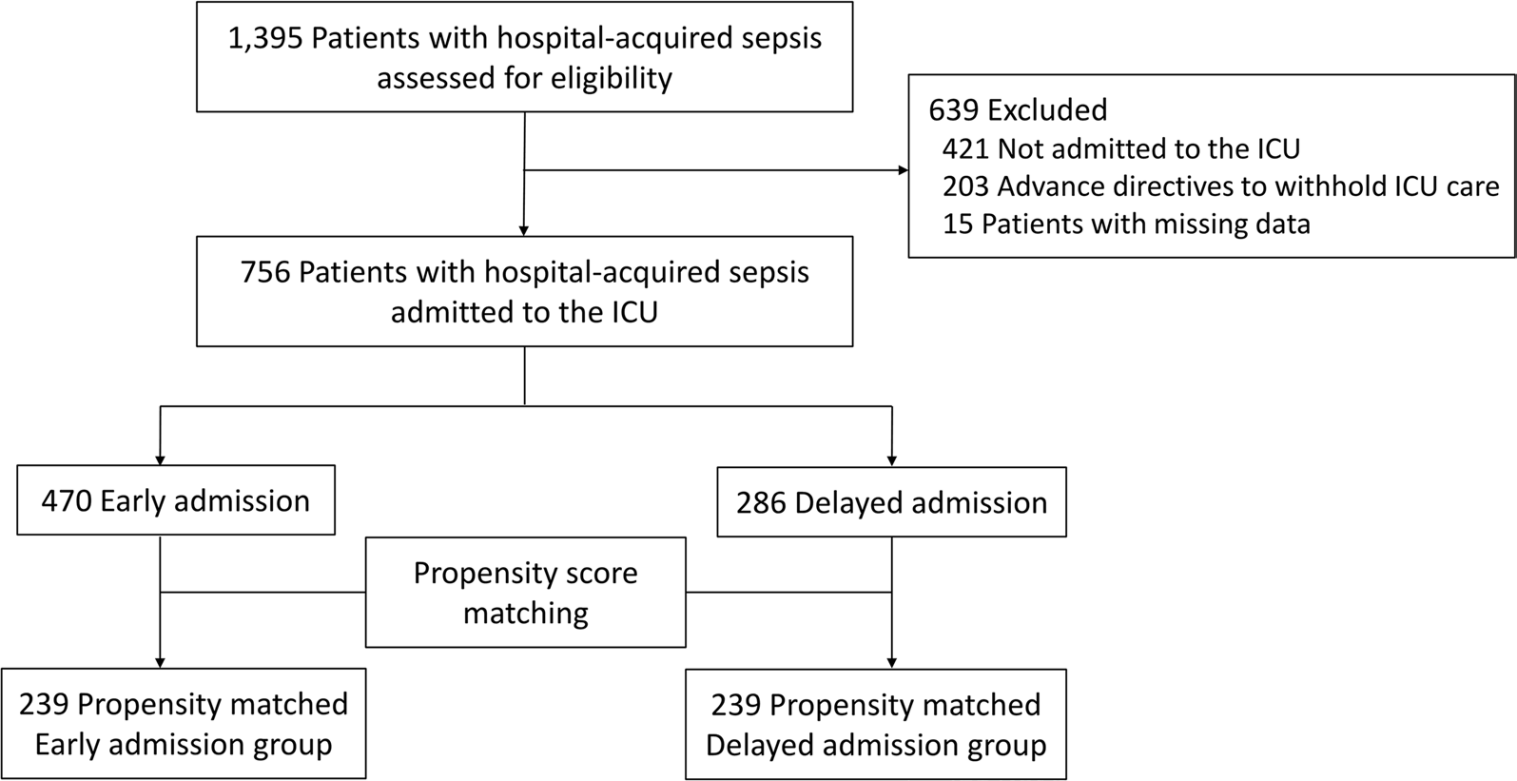
Study design. Early and delayed admission indicate admission to the ICU within 6 h or beyond 6 h, respectively. Hospital-onset sepsis was defined as sepsis diagnosed in the general ward

A total of 1395 patients with hospital-onset sepsis were assessed for eligibility between September 1, 2019, and December 31, 2020. Among these patients, 421 not admitted to the ICU, 203 with advance care directives to withhold life-sustaining treatment, and 15 patients with missing data were excluded. Of the patients admitted to the ICU, 756 with hospital-onset sepsis were included. Patients were categorized into two groups based on the timing of ICU admission: 470 patients belonged to the early admission group and 286 patients were included in the delayed admission group. After propensity score estimation, 239 matched pairs of patients were generated (Fig. 1).

The baseline characteristics of the study population are shown in Table 1. The median time to ICU admission was 1.9 h (0.8–3.4 h) for the early admission group in both cohorts; for the delayed group, the median times were 12.9 h (8.6–24.4 h) and 12.5 h (8.4–21.8 h) in the unmatched and matched cohorts, respectively. In the unmatched cohort, patients in the early admission group had significantly higher SOFA scores (7 [5–10] vs. 6 [4–9]; *P* < 0.001), higher incidence of shock at initial presentation (188 [40.0%] vs. 93 [32.5%]; *P* = 0.04), and greater lactate levels (3.2 [1.8–6.0] vs. 2.6 [1.7–4.3] mmol/L; *P* = 0.001). In the matched cohort, the mean age was 66 ± 14 years, 65.4% were men, and the mean BMI was 22.9 ± 4.3 kg/m^2^. The median SOFA score was 6 (4–9), the median lactate level was 2.6 mmol/L (1.7–4.5 mmol/L), and 37.3% of patients initially presented with septic shock. The SMDs were < 0.1 for all baseline characteristics, and the propensity score distributions shared common support for the covariates in the model, indicating a balance between the two groups (Additional file 1: Figure S1).

**Table 1:** Baseline characteristics according to the timing of ICU admission

Characteristic	Unmatched cohort			Propensity-Score-Matched cohort		
	Early admission (*N* = 470)	Delayed admission (*N* = 286)	SMD	Early admission (*N* = 239)	Delayed admission (*N* = 239)	SMD
Age—mean (SD)	66.6 ± 13.2	66.2 ± 14.2	0.03	66.5 ± 12.8	65.9 ± 14.1	0.04
Sex, male—no. (%)	306 (65.1)	189 (66.1)	0.02	163 (68.2)	154 (64.4)	0.08
Body mass index—mean (SD), kg/m^2^	22.9 ± 4.3	22.7 ± 4.1	0.05	23.0 ± 4.6	22.9 ± 4.0	0.02
Comorbidities—no. (%)						
Cardiovascular disease	90 (19.1)	80 (28.0)	0.21	58 (24.3)	53 (22.2)	0.05
Diabetes mellitus	180 (38.3)	106 (37.1)	0.03	89 (37.2)	83 (34.7)	0.05
Chronic liver disease	60 (12.8)	47 (16.4)	0.10	40 (16.7)	42 (17.6)	0.02
Chronic kidney disease	81 (17.2)	47 (16.4)	0.02	44 (18.4)	38 (15.9)	0.07
Solid malignancy	190 (40.4)	98 (34.3)	0.13	83 (34.7)	84 (35.1)	0.01
Hematologic malignancy	62 (13.2)	53 (18.5)	0.15	39 (16.3)	43 (18.0)	0.04
Chronic obstructive pulmonary disease	47 (10.0)	45 (15.7)	0.17	33 (13.8)	32 (13.4)	0.01
Clinical frailty scale—median (IQR)^a^	4 (3–6)	4 (3–7)	0.11	5 (3–7)	4 (3–7)	0.06
SOFA score—median (IQR)^b^	7 (5–10)	6 (4–9)	0.27	6 (4–10)	7 (4–9)	0.01
Septic shock—no. (%)^c^	188 (40.0)	93 (32.5)	0.16	85 (35.6)	90 (37.7)	0.04
Lactate—median (IQR), mmol/L^d^	3.2 (1.8–6.0)	2.6 (1.7–4.3)	0.34	2.5 (1.4–4.6)	2.7 (1.7–4.4)	0.01

Values are expressed as mean ± SD. ICU, intensive care unit; SMD, standardized mean difference; IQR, interquartile range

^a^The clinical frailty scale ranges from 1 to 9, with a score of 5 or greater indicating frailty

^b^Scores on the Sequential Organ Failure Assessment (SOFA) scale range from 0 to 24, with higher scores indicating more severe organ dysfunction

^c^Septic shock is defined as a vasopressor requirement to maintain the mean arterial pressure at or above 65 mmHg with a serum lactate level > 2 mmol/L

^d^Initial lactate levels at the time of sepsis diagnosis. Missing lactate values for 14 patients (3%) in the early admission group and 21 patients (7.3%) in the delayed admission group

### Primary outcome

Early admission to the ICU did not result in lower in-hospital mortality, with deaths reported in 181 of 470 patients (38.5%) in the early group and 119 of 286 (41.6%) in the delayed group (adjusted odds ratio [aOR], 1.35; 95% CI, 0.99–1.85). Similar results were observed in the matched cohort, with deaths occurring in 83 of 239 patients (34.7%) and 100 of 239 (41.8%) in the early and delayed admission groups, respectively (aOR, 1.38; 95% CI, 0.94–2.02) (Table 2). A sensitivity analysis exploring the effect of the center variable on the primary outcome also yielded comparable results (Additional file 1: Table S2). When treated as continuous data, the time to ICU admission was not significantly associated with increased odds of mortality at all timepoints (Additional file 1: Figure S2).

**Table 2:** In-hospital mortality according to the timing of ICU admission

Group	No. of deaths/total no. of patients (%)	*P* value	Unadjusted OR (95% CI)	Adjusted OR (95% CI)^a^
Unmatched cohort				
Early admission	181/470 (38.5)	0.44	1.14 (0.84–1.54)	1.35 (0.99–1.85)
Delayed admission	119/286 (41.6)			
Propensity-score-matched cohort				
Early admission	83/239 (34.7)	0.13	1.35 (0.93–1.96)	1.38 (0.94–2.02)
Delayed admission	100/239 (41.8)			

OR, odds ratio; CI, confidence interval

^a^In the unmatched cohort, odds ratios were adjusted for age and initial sequential organ failure assessment (SOFA) scores. In the propensity score-matched cohort, the odds ratios were adjusted for age, SOFA scores, and propensity scores

**Fig. 2:**
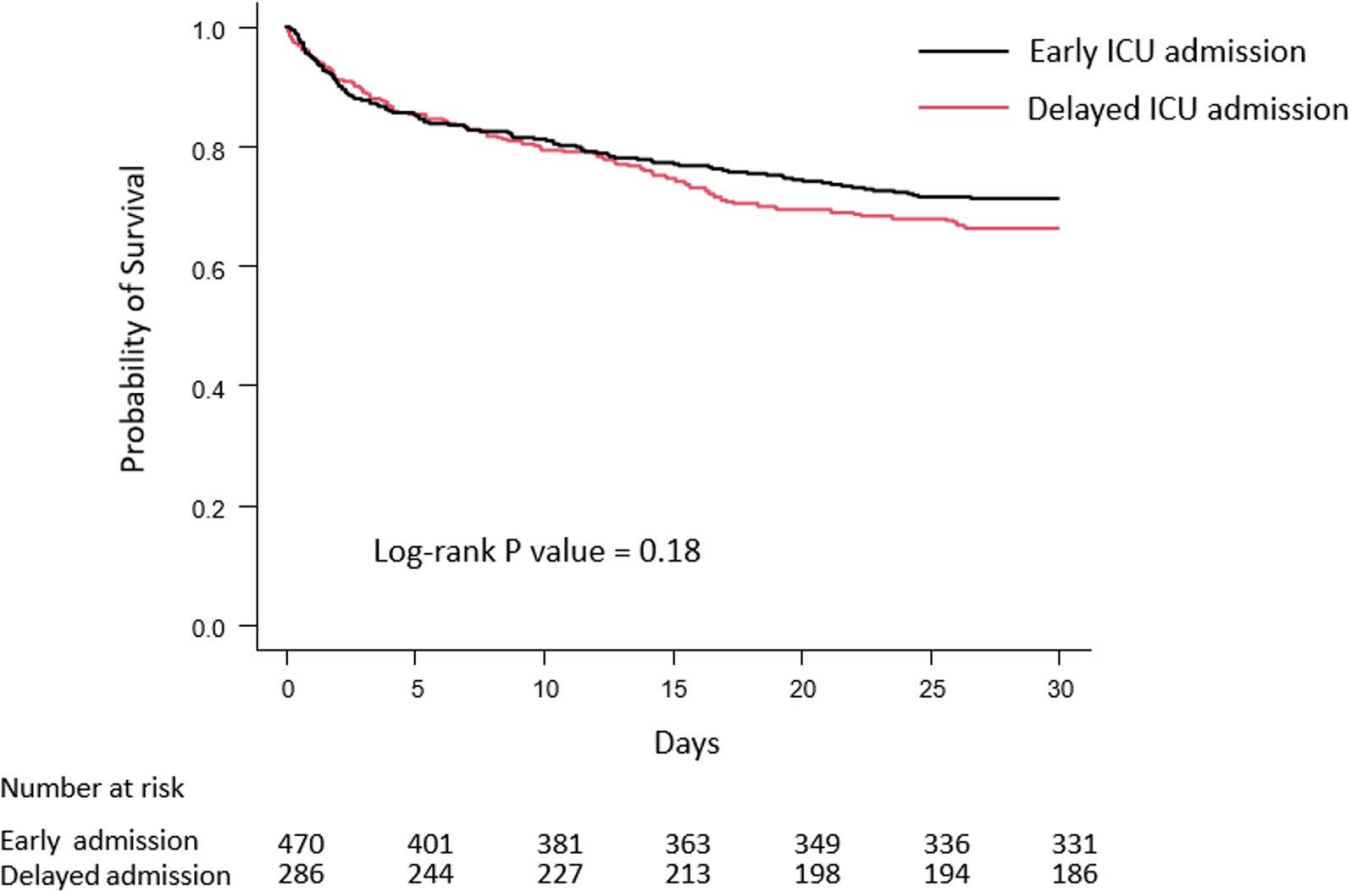
Kaplan–Meier estimates of 28-day mortality according to the timing of ICU admission. For each time interval, the survival probability was calculated as the number of patients who survived divided by the number of patients at risk

**Fig. 3:**
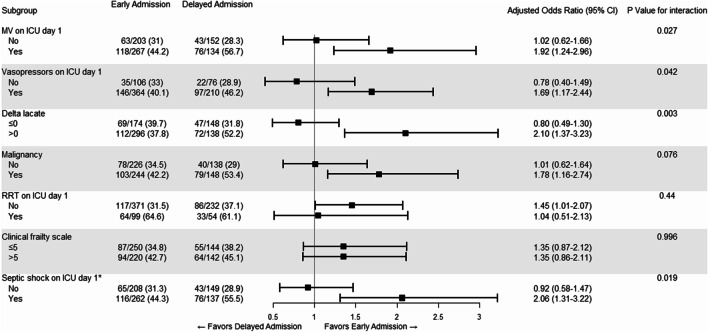
Odds ratios for the primary outcome in the prespecified subgroups. In-hospital mortality rates were compared between the early and delayed admission groups. Odds ratios were adjusted for age and the initial sequential organ failure assessment (SOFA) scores. ICU day 1 indicates the day of ICU admission. * Indicates post-hoc subgroup analysis

The Kaplan–Meier estimates of mortality in the two groups are shown in Fig. 2. The curves did not significantly diverge during the study period, and no difference in mortality was observed (*P* = 0.18, log-rank test). Prespecified subgroup analyses showed that patients who required mechanical ventilation on the day of ICU admission had a higher risk of in-hospital mortality with delayed admission (aOR, 1.92; 95% CI, 1.24–2.96; *P* for interaction = 0.027). Likewise, the risk of in-hospital mortality was higher in patients who needed vasopressor support on the day of ICU admission (aOR, 1.69; 95% CI, 1.17–2.44; *P* for interaction = 0.042) and in those with higher delta lactate levels (aOR, 2.10; 95% CI, 1.37–3.23; *P* for interaction = 0.003) (Fig. 3). A post-hoc subgroup analysis showed that patients with septic shock on the day of ICU admission also had a higher risk of in-hospital mortality with delayed admission (aOR, 2.06; 95% CI, 1.31–3.22; *P* for interaction = 0.019). No significant interactions were found in the other subgroups, including malignancy, RRT on the day of ICU admission, and clinical frailty status.

### Secondary outcomes

The secondary outcomes of hospital LOS, ICU LOS, and discharge location are summarized in Additional file 1: Table S3. There were no significant differences in the median hospital LOS (early admission, 20 [11–38] days; delayed admission, 18 [9–37] days; *P* = 0.44) and ICU LOS (early admission, 5 [2–12] days; delayed admission, 5 [2–10] days; *P* = 0.27) between the two groups. Of the 289 patients who survived in the early admission group, 211 (73.0%) were discharged to home, while 78 (27.0%) were discharged to another hospital or nursing facility. In the delayed admission group, 113 of 167 survivors (67.7%) were discharged to home, while 54 of 167 (32.3%) were discharged to another hospital or nursing facility. The discharge location was not significantly different between the two groups (*P* = 0.23). In addition, patients with advance care directives not admitted to the ICU had a significantly higher risk of in-hospital mortality, with deaths occurring in 148 of 203 patients (72.9%) with terminal diseases and in 300 of 756 (39.7%) patients without terminal diseases admitted to the ICU (*P* < 0.001). Details on the RRS of the participating hospitals and a description of the 1-h and 3-h sepsis bundle compliance are provided in Additional file 1: Figure S3, Table S4, and Table S5, respectively. Further details on the initial antibiotic therapy and source of infection are provided in Additional file 1: Table S6, Figure S4, and Figure S5, respectively.

## Discussion

In this nationwide study of patients with hospital-onset sepsis, admission to the ICU within 6 h did not significantly result in lower in-hospital mortality compared to delayed admission. We used propensity scores to adjust for confounding variables and illness severity [23], and still no significant differences in mortality were observed. Subgroup analyses showed that early ICU admission was associated with a lower risk of mortality in patients with septic shock, increasing lactate levels or those who needed vasopressor or ventilatory support on the day of ICU admission. To our knowledge, this study is the first to identify which patients with sepsis will benefit most from early admission to the ICU.

Our findings for the primary outcome conflict with those of a prospective cohort study of 401 critically ill patients in which a delay in ICU admission was associated with a higher mortality rate [5]. However, this study included patients not only diagnosed with sepsis but also those with intracranial hemorrhage, multiple trauma, and post-cardiac arrest. The management of these critical illnesses requires a multidisciplinary effort with a heavy dependency on organ support resources available only in the ICU. In contrast, the use of appropriate antibiotics to control the infection and mitigate its effects on organ dysfunction is the cornerstone of sepsis management [1], and antibiotics can be given anywhere, whether in the ED, wards, or the ICU. In the present study, there was no difference between the two groups in terms of adherence to the 1-h and 3-h bundle component of antibiotic administration. Thus, with timely administration of antibiotics and adequate source control, early admission to the ICU may not always be necessary to improve patient outcomes.

While some studies have demonstrated an association between mortality and delays in ICU admission, [24, 25] others have reported contradictory results. For example, a prospective cohort study of 1675 patients admitted to the ICU showed that the length of ED stay was not associated with hospital mortality (*P* = 0.82) [11]. Another case–control study of 358 patients with severe sepsis showed that compared with direct ICU admission, delayed admission was not independently associated with increased mortality (OR, 1.40; 95% CI, 0.73–2.76) [26]. The results of these studies are in agreement with our findings, and the conflicting nature of whether early ICU admission is beneficial for critically ill patients can perhaps be attributed to the heterogeneity of the patient population, differences in resource availability, and discrepancies in the quality of care provided outside the ICU [26].

Delays in ICU admission can be attributed to multiple factors. A study of 102 critically ill patients showed that a shortage of ICU beds (65.1%) and holdups in radiological examination (15.1%) were common reasons for delayed admission [27]. A meta-analysis conducted by Keikkas et al. [28] found that the main reasons for delayed ICU admission were an increase in the demand for ICU beds due to population aging and miscommunication among physicians; this study also found that patients with delayed admission tended to be older and had more comorbidities. Similarly, a study that investigated the association between the timing of ICU admission and outcomes in patients with pneumonia demonstrated that the decision to admit a patient to the ICU was often limited by the patient’s age, comorbidities, and premorbid functional status [29]. As the unequal distribution of patient characteristics may confound the association between the timing of ICU admission and mortality, we used propensity score matching as a way to adjust for such intrinsic factors. After balancing key covariates including age, comorbidities, and sepsis severity, we still found no significant association between admission to the ICU within 6 h and in-hospital mortality.

ICU beds are limited resources that require prioritization of admissions for sicker patients when demand exceeds supply [30]. A prospective cohort study of 1913 patients showed that the main factor responsible for  delayed transfers from the ED to the ICU was the ICU occupancy rate [31]. Harris and colleagues demonstrated that prompt admissions decreased as critical care bed occupancy increased [8]. Considering this pertinent issue of adequate resource allocation, identifying specific patient populations who will benefit from early ICU admission is important. Our prespecified subgroup and post-hoc analyses showed that patients with increasing lactate levels, those who needed vasopressor support, and septic shock on the day of ICU admission had a lower risk of mortality with early admission. These findings are unsurprising considering that serum lactate is a well-established marker of illness severity, [32] and that hypotension hinders oxygen delivery leading to multiorgan dysfunction [33]. Septic shock is characterized by decreased systemic vascular resistance, and higher vasopressor requirements have been found to reflect the severity of circulatory failure [34, 35]. In addition, delayed admission was associated with higher mortality in patients who required mechanical ventilation on the day of ICU admission. Previous studies have shown that patients with sepsis are more prone to lung injury during mechanical ventilation, and patient outcomes are influenced by whether optimized ventilatory support is provided efficiently [36–38]. Altogether, our findings emphasize the need for early, specialized care in certain patient populations.

Our study showed that delayed ICU admission was not associated with an increased hospital or ICU LOS. These findings are similar to those of a previous study of 2356 critically ill patients admitted to the ICU of a tertiary hospital, where 1595 (67.7%) were admitted beyond 6 h [39]. This study showed no significant difference in the median hospital LOS between the non-delayed and delayed admission groups (48 [22–96] vs. 67 [24–136] h; *P* = 0.46) [39]. Conversely, a retrospective cohort study of 1242 patients on mechanical ventilation in the ED showed that delayed ICU admission was associated with prolonged hospital stay (OR 1.56; 95% CI, 1.07–2.27) [38]. A major difference between these two studies was that only 12 patients (7.4%) in the delayed group of the former were on ventilatory support, while all patients in the latter required mechanical ventilation. Based on such findings, we suggest that critically ill patients who require mechanical ventilation be admitted to the ICU within 6 h, as delays in this subgroup of patients are associated with adverse outcomes.

Our study has several strengths. This nationwide, multicenter, prospective study included a specific subset of patients diagnosed with hospital-onset sepsis. While most previous studies analyzed a broad subset of critically ill patients, we performed a focused evaluation on patients with sepsis to provide evidence behind the SSC guideline that suggests that patients with sepsis or septic shock be admitted to the ICU within 6 h [4]. Through propensity score matching, we were able to consider multiple baseline risk factors for mortality based on the timing of ICU admission, thereby reducing the effects of confounding. Finally, we were able to show heterogeneity in increased mortality with delayed ICU admission in patients with increasing lactate levels, septic shock, and those who needed vasopressor or ventilatory support on the day of ICU admission. Resource allocation is crucial, especially in the wake of the coronavirus pandemic, where patients die at home waiting for a hospital bed [40]. In times like these, the findings of our study can help identify patients who will most likely benefit from early admission to the ICU.

Our study had several limitations. First, although possible confounders were addressed through propensity score matching, the risk of unmeasured confounders may still exist. Second, the effects of ICU admission timing on mortality may have been offset by a larger proportion of sicker patients in the early admission group within the entire cohort. However, we used propensity score matching to reduce the effects of confounder imbalance and found no differences in the primary outcome in both cohorts. Third, our data did not specify whether a physician or a nurse of the RRS made the sepsis diagnosis. Nevertheless, the qualifications of the RRS are stringent in South Korea: while physicians need to be board-certified in either internal medicine, neurology, general surgery, neurosurgery, cardiothoracic surgery, anesthesiology, or emergency medicine, nurses are required to have a minimum of 3 years of experience working in either an ICU or the emergency department in order qualify as a member of the RRS [41]. As such, all members of the RRS at each participating hospital were highly trained to accurately recognize and diagnose sepsis. Finally, as our data did not include information about ICU bed availability and delays in radiological examination, we were unable to analyze how such factors influenced the timing of ICU admission.

## Conclusion

Among patients with hospital-onset sepsis, in-hospital mortality did not differ significantly between those with early and delayed ICU admission. However, admission to the ICU within 6 h may benefit those with increasing lactate levels, septic shock, and those who require vasopressors or ventilatory support. Thus, early ICU admission should be considered for these subsets of patients.

## Supplementary Information

**Additional file 1.** Supplementary figures and tables.

## Data Availability

The data sets used and/or analyzed during the current study are available from the corresponding author on reasonable request.

## Abbreviations

BMI: Body mass index
CI: Confidence interval
ED: Emergency department
ICU: Intensive care unit
IQR: Interquartile range
IRB: Institutional review board
LOS: Length of stay
MAP: Mean arterial pressure
MV: Mechanical ventilation
OR: Odds ratio
RRS: Rapid response system
RRT: Renal replacement therapy
SD: Standard deviation
SMD: Standardized mean difference
SOFA: Sequential organ failure assessment
