# Supplementary material for: Association between the timing of ICU admission and mortality in patients with hospital-onset sepsis: a nationwide prospective cohort study
Source: J Intensive Care. 2023 Apr 21;11:16. doi: 10.1186/s40560-023-00663-6 (PMC10120484; doi:10.1186/s40560-023-00663-6)
Supplement: Supplementary file 1 — Additional file 1. Supplementary figures and tables. [file 40560_2023_663_MOESM1_ESM.pdf]

Additional File 1

Association between the timing of ICU admission and mortality in patients with hospital-onset sepsis: a nationwide prospective cohort study

Authors:

Yoon Hae Ahn, MD[1], Jinwoo Lee, MD, PhD[2], Dong Kyu Oh, MD, PhD[3], Su Yeon Lee, MD[3], Mi Hyeon Park[3], Haein Lee[3], Chae-Man Lim, MD, PhD[3], Sang-Min Lee, MD, PhD[1,2], Hong Yeul Lee, MD, PhD[1], on behalf of the Korean Sepsis Alliance (KSA) Investigators

Affiliations:

[1] Department of Critical Care Medicine, Seoul National University Hospital, Seoul, Korea
[2] Division of Pulmonary and Critical Care Medicine, Department of Internal Medicine, Seoul National University Hospital, Seoul, Korea
[3] Department of Pulmonary and Critical Care Medicine, Asan Medical Center, Seoul, Korea

Corresponding author:

Hong Yeul Lee, M.D.
Assistant Professor
Department of Critical Care Medicine, Seoul National University Hospital, 101 Daehak-ro, Jongno-gu, Seoul, 03080, Korea.
Tel: 82-2-2072-1457; Fax: 82-2-762-9662
E-mail: takumama@naver.com

Table of Contents

Figure S1……………………………………………………………………………………….3

Figure S2………………………………………………………………………………………4

Figure S3……………………………………………………………………………………….5

Figure S4………………………………………………………………………………………6

Figure S5………………………………………………………………………………………7

Table S1……..…………………………………………………………………………………8

Table S2……..……………………………………………………………………………...12

Table S3……..……………………………………………………………………………...13

Table S4……..……………………………………………………………………………...14

Table S5……..……………………………………………………………………………...15

Table S6……..……………………………………………………………………………...16

Figure S1. Distribution of the Propensity Score Before (left) and After (right) Matching

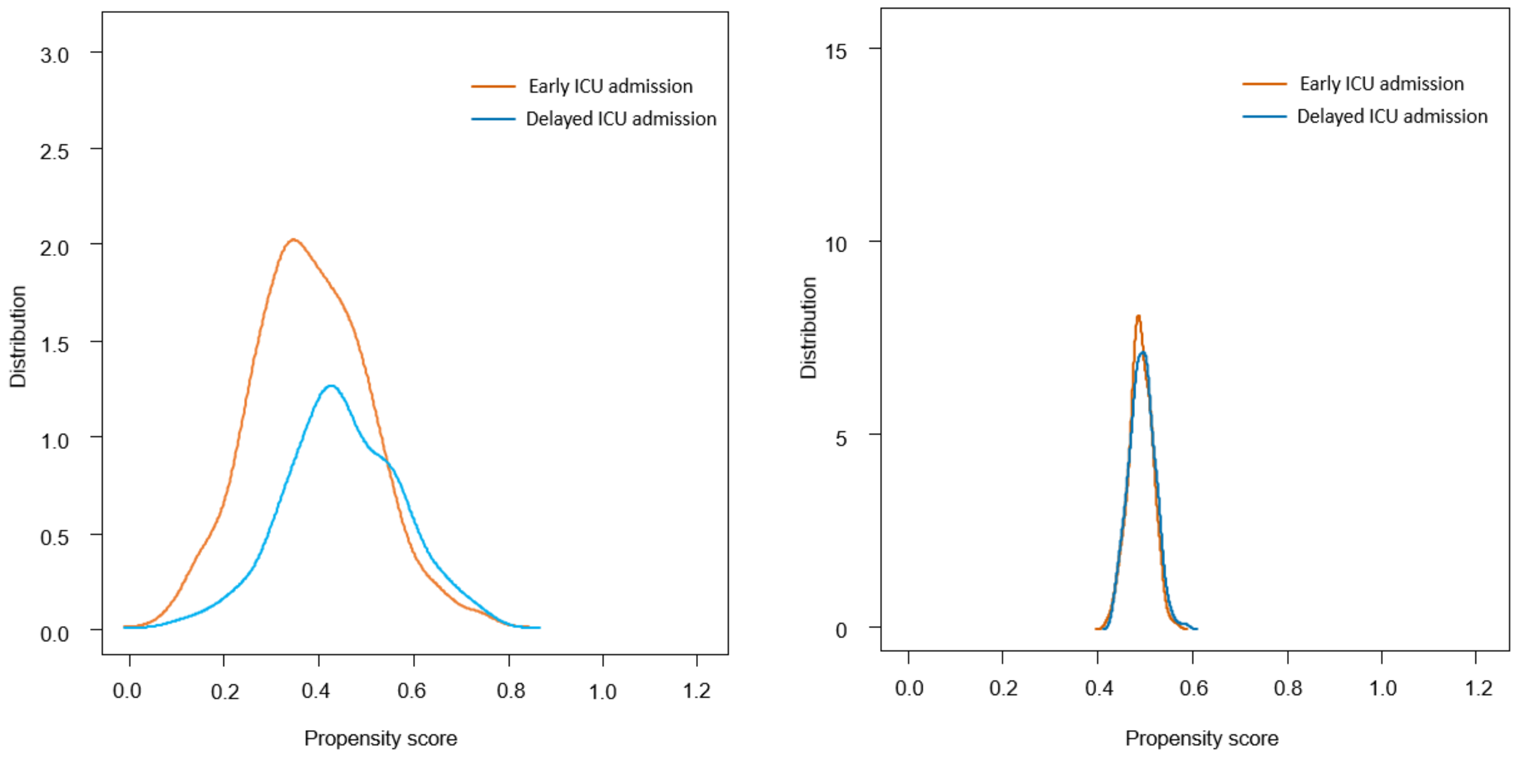

Figure S2. Time to ICU admission and In-hospital Mortality

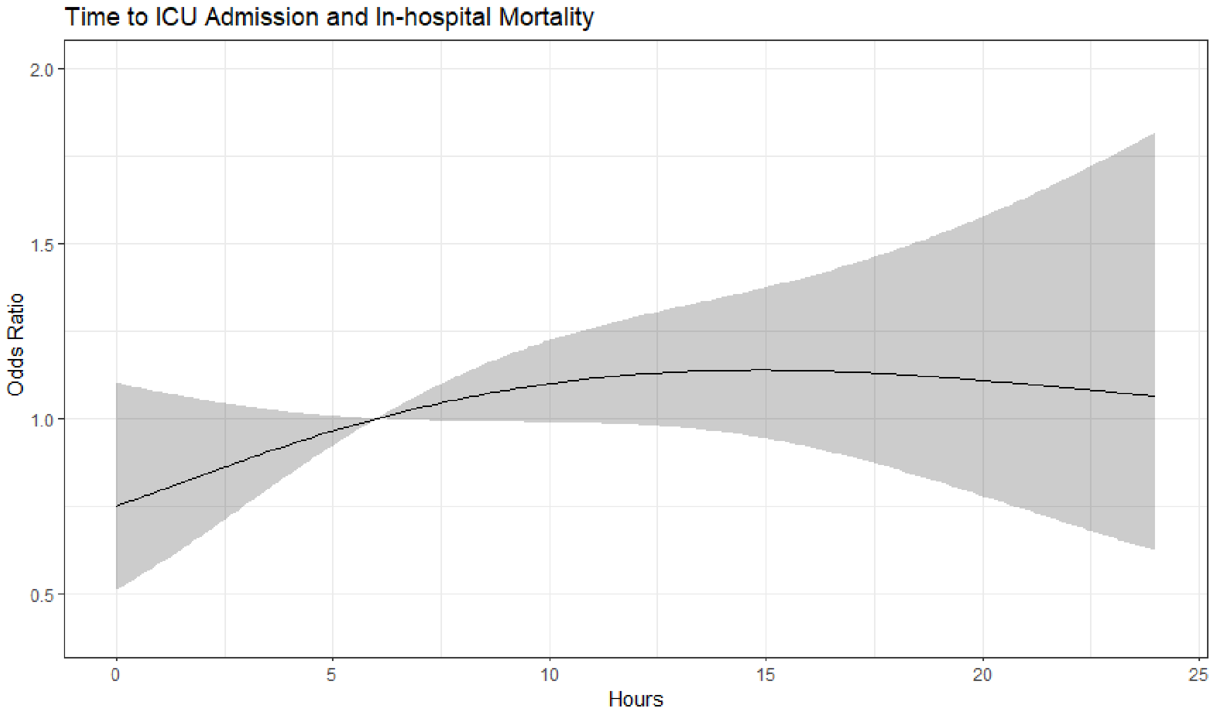

Figure S2. Restricted cubic spline plot of the association between the time to ICU admission and in-hospital mortality
The plot was adjusted for age and initial sequential organ failure assessment (SOFA) scores. The shaded areas represent the 95% confidence intervals (CI).

Figure S3. Rapid Response System by Operating Hours

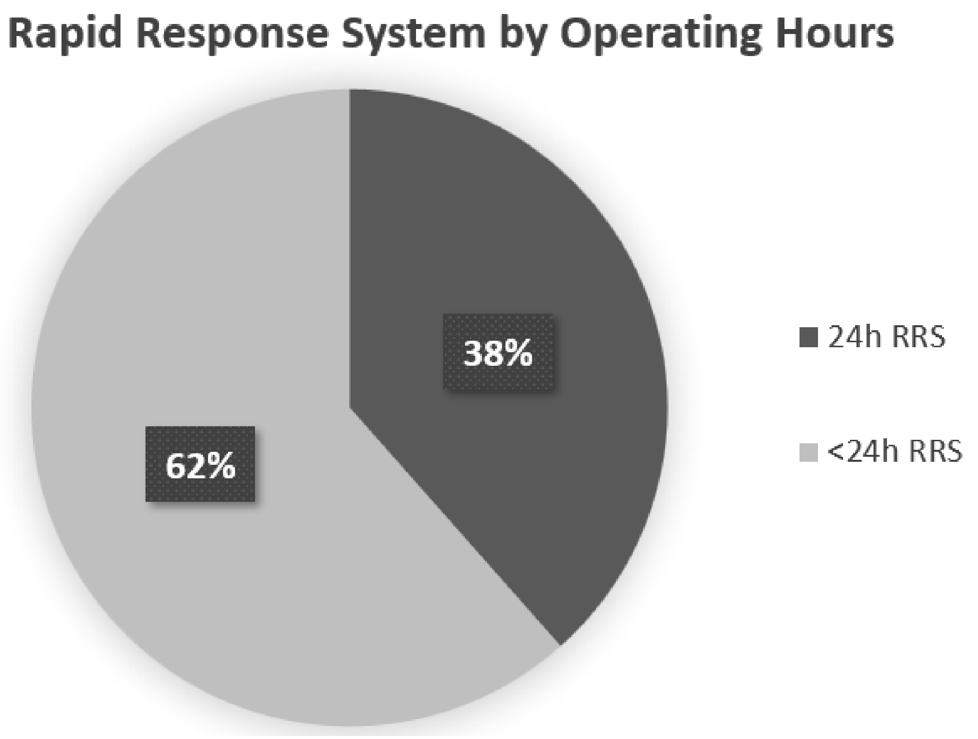

Figure S4. Initial Antibiotic Therapy at Each Hospital

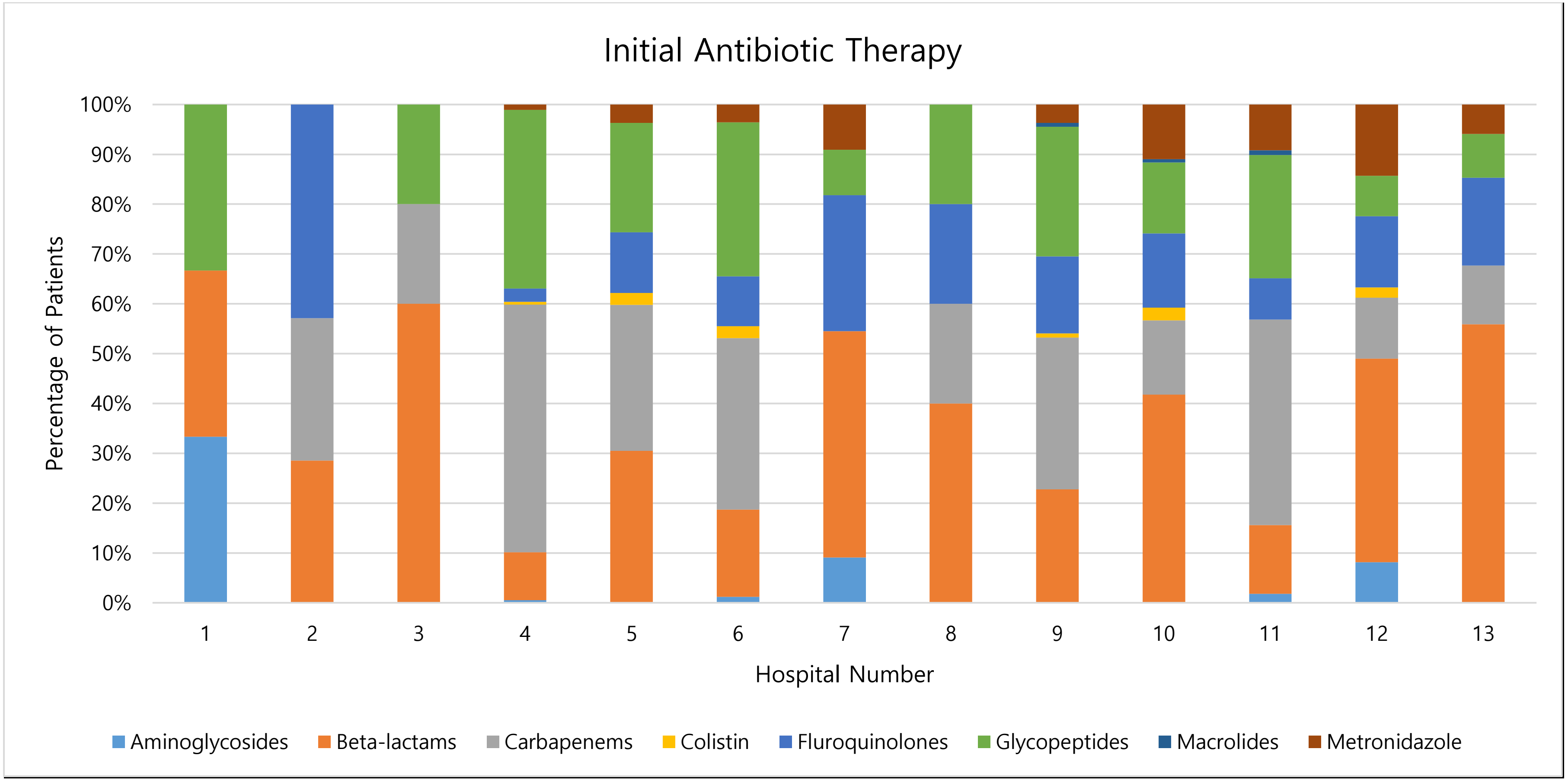

Figure S5. Source of Infection at Each Hospital

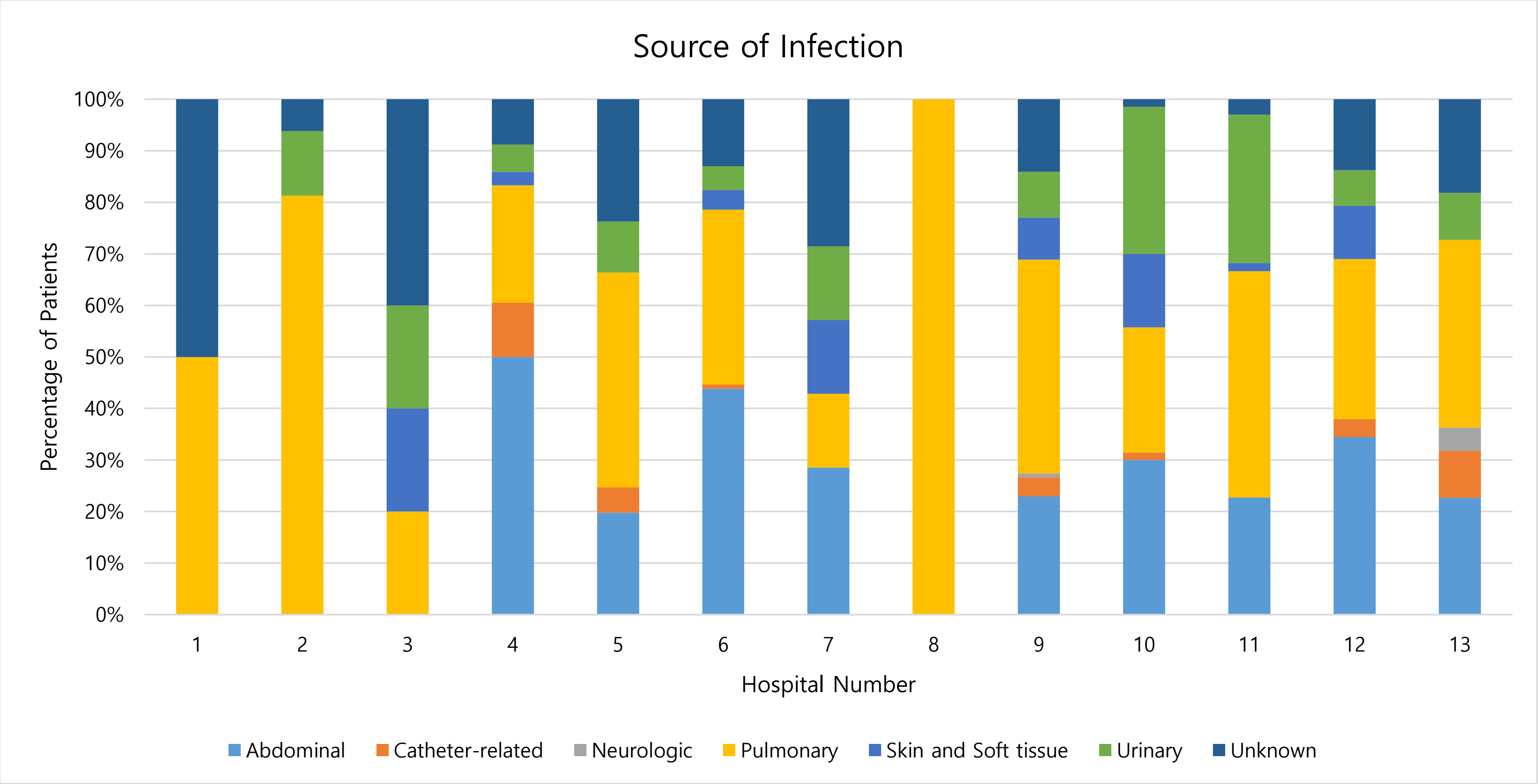

Table S1. Activation Criteria of Rapid Response System at Participating Hospitals

Center	Activation criteria
Korea University Anam Hospital	A. Screening criteria<br>- MEWS ≥ 2
Seoul National University Bundang Hospital	- Systolic blood pressure: <90 mmHg<br>- Heart rate: <50, >140/min<br>- Respiratory rate: <10, >30/min<br>- Body temperature: <36°C, >39°C<br>- SpO_2: <90%<br>- ABG: pH <7.25, $PaCO_2$ >50 mmHg, PaO_2 <55 mmHg<br>- Lactate >4 mmol/L<br>- Total CO_2 <15 mmol/L
Samsung Medical Center	A. Calling criteria<br>- Airway: compromised, stridor<br>- Respiratory: respiratory rate ≥30, SpO_2 <85% for 5 min, pH <7.3 and $PaCO_2$ >50 mmHg, use of accessory respiratory muscles<br>- Cardiovascular: systolic blood pressure <85 mmHg, heart rate >130, acute chest pain, symptomatic arrhythmia<br>- Neurologic: altered mental status of sudden onset, unexplained agitation, seizure<br>- Others: subjective assessment of physician or nurse<br>B. Screening criteria<br>- MEWS ≥7
Seoul National University Hospital	A. Calling criteria<br>- Respiratory: respiratory rate ≤ 8 or ≥28, SpO_2 ≤ 90% for 5 minutes, sudden onset dyspnea<br>- Cardiovascular: heart rate ≤ 40 or ≥130, systolic blood pressure ≤ 80 or ≥200, systolic blood pressure 80–90 mmHg with symptoms, chest pain unresponsive to sublingual nitroglycerin<br>- Neurologic: sudden onset of altered consciousness, sudden paralysis, new onset seizure, prolonged agitation (≥10 minutes) not fully explained by medical conditions<br>- Others: color change of peripheral extremities, subjective assessment of physician or nurse<br>B. Screening criteria<br>- Heart rate: <41, >129/min<br>- Respiratory rate: <9, >27/min<br>- Systolic blood pressure: <81, >199 mmHg<br>- SpO_2: <90%
Asan Medical Center	A. Calling criteria<br>- Airway: compromised, stridor

	- Respiratory: respiratory rate <6 or >30/min, SpO_2 <90% on high flow FiO2 ≥40% or O_2 ≥6 L/min<br>- Cardiovascular: heart rate <40 or >140/min, systolic blood pressure <90 mmHg<br>- Neurologic: sudden change in mental status, seizure<br>- Others: subjective assessment of attending physician or nurse<br>B. Screening criteria<br>- Systolic blood pressure <86 mmHg<br>- Sudden change in mental status or unexplained agitation<br>- Hypoxemia with oxygen requirement of >9 L/min or FiO_2 > 35%<br>- Respiratory rate >27 or <8/min<br>- Heart rate >140 or <40<br>- Unexplained severe metabolic acidosis: pH <7.3, lactate >2 mmol/L, total CO_2 <12 mmol/L<br>- $PaCO_2$ > 50 mmHg or PaO_2 ≤ 55 mmHg<br>- Subjective assessment of attending physician or nurse
Ulsan University Hospital	A. Calling criteria<br>- Airway: compromised, stridor, wheezing<br>- Respiratory: respiratory rate ≤6 or ≥30/min, SpO_2 <90% on O_2 ≥6 L/min or high flow FiO2 ≥40%<br>- Cardiovascular: heart rate < 40 or ≥140/min, systolic blood pressure < 90 mmHg<br>- Neurologic: sudden change in mental status, seizure<br>- Others: subjective assessment of attending physician or nurse<br>B. Screening criteria<br>- Airway: compromised, stridor, wheezing<br>- Breathing: respiratory rate ≤6 or ≥30/min, SpO_2 <90% on O_2 ≥6 L/min or high flow FiO2 ≥40%<br>- Cardiovascular: heart rate < 40 or ≥140/min, systolic blood pressure ≤ 85 mmHg<br>- Neurologic: sudden change in mental status, seizure<br>- Others: pH ≤7.33, HCO_3 ≤15 mmol/L, lactate ≥2 mmol/L, potassium ≥6 mmol/L, total CO_2 ≤15 mmol/L, glucose ≤50 mg/dL, patients recently discharged from the ICU
Chonnam National University Hospital	- Systolic blood pressure <80 mmHg, heart rate <40 or >140/min, respiratory rate ≤10 or ≥ 30/min, SpO_2 ≤85%<br>- ABG: pH <7.3, pCO_2 >50 mmHg, pO_2 <60 mmHg<br>- Sudden change in mental status<br>- Symptomatic arrhythmia
Jeonbuk National University Hospital	A. Screening criteria<br>- NEWS ≥ 5

Chungnam National University Hospital	A. Calling criteria<br>- Airway: obstruction, stridor<br>- Respiratory: respiratory rate <8 or >30/min, pH <7.3 and $PaCO_2$ > 60 mmHg, SpO_2 <90% on facial mask or high flow nasal cannula<br>- Cardiovascular: heart rate <40 or >140/min, systolic blood pressure <90 mmHg, lactate >2 mmol/L<br>- Neurologic: sudden change in mental status, unexplained agitation, seizure<br>- Subjective assessment of attending physician or nurse<br>B. Screening criteria<br>- based on NEWS
Chungnam National University Sejong Hospital	A. Calling criteria<br>- Airway: obstruction, stridor<br>- Respiratory: respiratory rate <8 or >30/min, pH <7.3 and $PaCO_2$ > 60 mmHg, SpO_2 <90% on facial mask or high flow nasal cannula<br>- Cardiovascular: heart rate <40 or >140/min, systolic blood pressure <90 mmHg, lactate >2 mmol/L<br>- Neurologic: sudden change in mental status, unexplained agitation, seizure<br>- Subjective assessment of attending physician or nurse<br>B. Screening criteria<br>- based on NEWS
Hanyang University Guri Hospital	A. Calling criteria<br>- Airway: airway obstruction sign, stridor<br>- Respiratory: respiratory rate <8 or >30/min, pH <7.3 and $PaCO_2$ >60 mmHg, SpO_2 <90% on facial mask or high flow nasal cannula<br>- Cardiovascular: heart rate <40 or >140/min, systolic blood pressure <90 mmHg, lactate >2 mmol/L<br>- Neurologic: sudden change in mental status, unexplained agitation, seizure<br>- Subjective assessment of attending physician or nurse<br>B. Screening criteria<br>- MEWS ≥4<br>- Systolic blood pressure ≤80 or ≥200 mmHg, heart rate ≤40 or ≥130/min, respiratory rate <9 or ≥30/min, SpO_2 ≤90%<br>- ABG: pH ≤7.3, pCO_2 ≥50 mmHg, pO_2 ≤60 mmHg, HCO_3 ≤14 mmEq/L, lactate ≥4.0 mmol/L<br>- Chemistry: potassium ≥6.0 mmol/L, total CO_2 ≤14 mmol/L
Daegu Catholic University Hospital	A. Calling criteria<br>- Respiratory: respiratory rate <8 or ≥30/min, pH <7.3 and $PaCO_2$ >60 mmHg, SpO_2 <90% on facial mask<br>- Cardiovascular: heart rate <40 or ≥140/min, systolic blood pressure <80 mmHg<br>- Neurologic: sudden mental change, unexplained agitation for ≥10 minutes<br>- Subjective assessment of attending physician or nurse<br>B. Screening criteria<br>- NEWS ≥ 5

Yeungnam University Medical Center	A. Screening criteria<br>- NEWS ≥5<br>- Respiratory rate <10 or ≥30/min<br>- Heart rate <40 or ≥140/min<br>- Systolic blood pressure ≤80 mmHg<br>- SpO_2 ≤90%

ABG, Arterial blood gas analysis; FiO_2, fraction of inspired oxygen; MEWS, modified early warning score; NEWS, national early warning score; $PaCO_2$, partial pressure of carbon dioxide in arterial blood; PaO_2, partial pressure of oxygen in arterial blood; pCO_2, partial pressure of carbon dioxide; pO_2, partial pressure of oxygen; SpO_2, oxygen saturation

Table S2. In-hospital Mortality According to the Timing of ICU admission

Group	No. of deaths / Total no. of patients (%)	*P*-value	Unadjusted OR (95% CI)	Adjusted OR (95% CI)[a]
**Unmatched cohort**				
Early admission	181/470 (38.5)	0.44	1.14 (0.84–1.54)	1.36 (0.99–1.87)
Delayed admission	119/286 (41.6)			
**Propensity-score-matched cohort**				
Early admission	83/239 (34.7)	0.13	1.35 (0.93–1.96)	1.38 (0.94–2.03)
Delayed admission	100/239 (41.8)			

OR, odds ratio; CI, confidence interval

[a] In the unmatched cohort, odds ratios were adjusted for age and initial sequential organ failure assessment (SOFA) scores and center variables. In the propensity score-matched cohort, the odds ratios were adjusted for age, SOFA scores, center variables and propensity scores.

Table S3. Secondary Outcomes According to the Timing of ICU Admission

Outcome	Early admission (N = 470)	Delayed admission (N = 286)	*P*-value
**Median hospital length of stay (IQR), days**			
In-hospital survivors	27 (16–46)	26 (15–48)	0.93
In-hospital non-survivors	10 (2–29)	12 (4–24)	0.21
**Median ICU length of stay (IQR), days**			
ICU survivors	5 (3–11)	4 (2–9)	0.35
ICU non-survivors	4 (2–14)	5 (2–12)	0.30
**Discharge, n/total (%)[a]**			0.23
At home	211/289 (73.0)	113/167 (67.7)	
Transfer at another hospital	78/289 (27.0)	54/167 (32.3)	

ICU, intensive care unit; IQR, interquartile range
[a] Analysis was performed only in patients who survived until discharge

Table S4. Sepsis Bundle Compliance According to the Timing of ICU Admission

Variable	Unmatched cohort			Propensity Score-Matched Cohort		
	Early admission (N = 470)	Delayed admission (N = 286)	*P*-value	Early admission (N = 239)	Delayed admission (N = 239)	*P*-value
**1-Hour Bundle Compliance**						
Lactate measurement	273 (58.1)	134 (46.9)	0.003	142 (59.4)	121 (50.6)	0.066
Blood cultures	285 (60.6)	170 (59.4)	0.760	137 (57.3)	147 (61.5)	0.402
Administration of antibiotics[a]	172/368 (46.7)	104/248 (41.9)	0.248	85/185 (45.9)	85/208 (40.9)	0.359
Crystalloid infusion[b]	205/283 (72.4)	111/138 (80.4)	0.093	99/135 (73.3)	99/126 (78.6)	0.385
Vasopressor use[c]	167/279 (59.9)	64/134 (47.8)	0.026	78/135 (57.8)	62/122 (50.8)	0.316
**3-Hour Bundle Compliance**						
Lactate measurement	344 (73.2)	169 (59.1)	<0.001	179 (74.9)	153 (64.0)	0.013
Blood cultures	359 (76.4)	202 (70.6)	0.087	176 (73.6)	175 (73.2)	1.000
Administration of antibiotics[a]	260/368 (70.7)	160/248 (64.5)	0.113	130/185 (70.3)	135/208 (64.9)	0.282
Crystalloid infusion[b]	208/283 (73.5)	112/138 (81.2)	0.090	101/135 (74.8)	100/126 (79.4)	0.462
Vasopressor use[c]	219/279 (78.5)	85/134 (63.4)	0.002	102/135 (75.6)	78/122 (63.9)	0.056

[a] Initiation of antibiotic therapy on patients who were not previously on antibiotics
[b] Patients with either hypotension (mean blood pressure < 65 mmHg) or lactate ≥ 4 mmol/L
[c] Patients with hypotension despite fluid resuscitation

Table S5. Time to ICU Admission by Rapid Response System Operating Hours

Outcome	24-Hour RRS (N = 446)	Part-time RRS[a] (N = 310)	*P*-value
**Time to ICU Admission (IQR), hours**	3.8 (1.6–9.5)	3.4 (1.1–10.4)	0.22
**Timing of ICU Admission, n (%)**			
Early Admission	274 (61.4%)	196 (63.2%)	0.67
Delayed Admission	172 (38.6%)	114 (36.8%)	

RRS, rapid response system; ICU, intensive care unit; IQR, interquartile range
[a] RRS that operated <24 hours

Table S6. Initial Antibiotic Therapy and Source of Infection According to the Timing of ICU Admission

Variable	Early admission (N = 470)	Delayed admission (N = 286)	*P*-value
**Initial Antibiotic Therapy**			
Aminoglycoside	12 (2.6)	3 (1)	0.19
Beta-lactam	201 (42.8)	124 (43.4)	0.88
Carbapenem	280 (59.6)	175 (61.2)	0.70
Colistin	12 (2.6)	10 (3.5)	0.51
Fluroquinolone	106 (22.6)	59 (20.6)	0.59
Glycopeptide	240 (51.1)	126 (44.1)	0.07
Macrolide	1 (0.2)	3 (1)	0.16
Metronidazole	44 (9.4)	25 (8.7)	0.80
**Source of Infection**			
Abdominal	174 (37)	92 (32.2)	0.18
Catheter-related	14 (3)	12 (4.2)	0.41
Neurologic	0 (0)	2 (0.7)	0.14
Pulmonary	169 (36)	104 (36.4)	0.94
Skin and soft tissue	27 (5.7)	13 (4.5)	0.51
Urinary	50 (10.6)	32 (11.2)	0.81
Systemic infections with unknown focus	48 (10.2)	44 (15.4)	0.04

ICU, intensive care unit
